# Efficacy and Safety of Tranexamic Acid in Prehospital Traumatic Hemorrhagic Shock: Outcomes of the Cal-PAT Study

**DOI:** 10.5811/westjem.2017.2.32044

**Published:** 2017-04-19

**Authors:** Michael M. Neeki, Fanglong Dong, Jake Toy, Reza Vaezazizi, Joe Powell, Nina Jabourian, Alex Jabourian, David Wong, Richard Vara, Kathryn Seiler, Troy W. Pennington, Joe Powell, Chris Yoshida-McMath, Shanna Kissel, Katharine Schulz-Costello, Jamish Mistry, Matthew S. Surrusco, Karen R. O’Bosky, Daved Van Stralen, Daniel Ludi, Karl Sporer, Peter Benson, Eugene Kwong, Richard Pitts, John T. Culhane, Rodney Borger

**Affiliations:** * Arrowhead Regional Medical Center, Department of Emergency Medicine, Colton, California; † Western University of Health Sciences, College of Osteopathic Medicine of the Pacific, Pomona, California; ‡ Arrowhead Regional Medical Center, Department of General Surgery, Colton, California; § Inland Counties Emergency Medical Agency, San Bernardino, California; ¶ California University of Sciences and Medicine, Colton, California; || City of Rialto Fire Department, Rialto, California; # Riverside County Emergency Services Agency, Riverside, California; ** Loma Linda University Medical Center, Department of General Surgery, Loma Linda, California; †† Loma Linda University Children’s Hospital, Department of Pediatrics, Loma Linda, California; ‡‡ Alameda County EMS Agency, San Leandro, California; §§ Napa County EMS Agency, Napa, California

## Abstract

**Introduction:**

The California Prehospital Antifibrinolytic Therapy (Cal-PAT) study seeks to assess the safety and impact on patient mortality of tranexamic acid (TXA) administration in cases of trauma-induced hemorrhagic shock. The current study further aimed to assess the feasibility of prehospital TXA administration by paramedics within the framework of North American emergency medicine standards and protocols.

**Methods:**

This is an ongoing multi-centered, prospective, observational cohort study with a retrospective chart-review comparison. Trauma patients identified in the prehospital setting with signs of hemorrhagic shock by first responders were administered one gram of TXA followed by an optional second one-gram dose upon arrival to the hospital, if the patient still met inclusion criteria. Patients administered TXA make up the prehospital intervention group. Control group patients met the same inclusion criteria as TXA candidates and were matched with the prehospital intervention patients based on mechanism of injury, injury severity score, and age. The primary outcomes were mortality, measured at 24 hours, 48 hours, and 28 days. Secondary outcomes measured included the total blood products transfused and any known adverse events associated with TXA administration.

**Results:**

We included 128 patients in the prehospital intervention group and 125 in the control group. Although not statistically significant, the prehospital intervention group trended toward a lower 24-hour mortality rate (3.9% vs 7.2% for intervention and control, respectively, p=0.25), 48-hour mortality rate (6.3% vs 7.2% for intervention and control, respectively, p=0.76), and 28-day mortality rate (6.3% vs 10.4% for intervention and control, respectively, p=0.23). There was no significant difference observed in known adverse events associated with TXA administration in the prehospital intervention group and control group. A reduction in total blood product usage was observed following the administration of TXA (control: 6.95 units; intervention: 4.09 units; p=0.01).

**Conclusion:**

Preliminary evidence from the Cal-PAT study suggests that TXA administration may be safe in the prehospital setting with no significant change in adverse events observed and an associated decreased use of blood products in cases of trauma-induced hemorrhagic shock. Given the current sample size, a statistically significant decrease in mortality was not observed. Additionally, this study demonstrates that it may be feasible for paramedics to identify and safely administer TXA in the prehospital setting.

## INTRODUCTION

Trauma accounts for more than five million deaths worldwide annually, equating to 9% of total world mortality.^1^ In the United States, traumatic injury is the leading cause of death among individuals aged 1 to 44 years old.^2^ The direct economic burden as a result of trauma is substantial in the U.S. In 2010, costs associated with unintentional traumatic injury exceeded $113 billion, including both medical and work-loss associated costs.^3^

Following acute traumatic injury, significant blood loss threatens the body’s ability to maintain hemodynamic stability. Nearly 25% of patients arriving to the emergency department (ED) present with an acute coagulopathy that may complicate management.^4^,^5^ Up to 40% of mortality due to trauma-related injuries results from hemorrhagic shock.^6^,^7^ Further, mortality secondary to hemorrhagic shock represents the largest fraction of deaths, both within the prehospital setting and within the first hour of trauma care.^6^ Historically, paramedics have not had access to medications that specifically assist in the treatment of hemorrhagic shock secondary to trauma.^6^,^8^ However, evidence suggests that early treatment of acute coagulopathies and hemorrhagic shock may significantly reduce preventable death.^6^,^9^–^11^

TXA, an antifibrinolytic agent, has been evaluated in two previous large-scale studies for the treatment of trauma-induced hemorrhagic shock. In 2010 the “Clinical Randomization of an Anti-fibrinolytic in Significant Hemorrhage 2” (CRASH-2) trial, was conducted in the civilian international setting and assessed the impact of TXA administration in patients with signs of hemorrhagic shock on trauma-related death, occlusive events and blood product transfusions. CRASH-2 demonstrated the potential effectiveness of TXA for use in trauma-related injuries with a 1.5% reduction in all-cause mortality at 28 days.^12^ TXA was also determined to significantly reduce the risk of death due to bleeding, both immediately after injury and at 28 days.^12^ In 2011 a subgroup analysis of the CRASH-2 trial showed that early treatment in the hospital setting with TXA, less than one hour from the time of injury, resulted in a 2.4% decrease in death due to bleeding.^13^ Another CRASH-2 economic subset analysis highlighted the fact that using TXA can be highly cost effective.^14^

In 2012 the “Military Application of TXA in Trauma Emergency Resuscitation” (MATTERs) study evaluated TXA administration in patients receiving at least one unit of packed red blood cells. Results suggested that hospital administration of TXA reduced all-cause mortality in comparison to those not administered TXA (17.4% vs 23.9%, respectively; p = .03).^15^ From these two large investigations, it appears that TXA may show potential benefit in the treatment of hemorrhagic shock.

In previous studies, TXA was primarily administered within the hospital setting.^12^,^15^ Two small studies have demonstrated the feasibility of TXA administration in the prehospital setting.^16^,^17^ However, both studies were based on a smaller sample size of 40 and 13 patients, which limited the generalizability of their findings.^16^,^17^ The goal of the California Prehospital Antifibrinolytic Therapy (Cal-PAT) study is to assess the safety and impact on mortality of prehospital TXA administration by paramedics in cases of traumatic injury with signs of hemorrhagic shock. The ultimate goal is to provide reliable evidence to support TXA utilization in the prehospital setting. This preliminary report from the ongoing Cal-PAT study assessed mortality impact, total blood product usage, and incidence of known side effects associated with the use of TXA. Further, this study evaluated paramedic ability to accurately identify TXA candidates and effectively administer TXA within the framework of North American emergency medicine standards and protocols. Though previous large-scale studies were completed in the civilian international setting and combat setting, this study intended to address TXA administration within the protocols set forth by United States EMS agencies, including current paramedic training standards and response paradigms.^12^,^15^

**Population Health Research Capsule**

What do we already know about this issue?

Prior studies assessing tranexamic acid (TXA) use in civilian and military trauma demonstrate a promising effect on mortality reduction and a limited side-effect profile.

What was the research question?

What is the impact and feasibility of prehospital TXA use in trauma-induced hemorrhagic shock within the framework of North American EMS standards?

What was the major finding of the study?

TXA use was associated with a decrease in blood product use and no apparent change in adverse events in traumatic hemorrhagic shock.

How does this improve population health?

Traumatic injury is a major cause of death in both developed and developing nations. TXA represents a cost-effective measure that may reduce loss of life due to exsanguinating injury.

## METHODS

### Cal-PAT Study Overview

The Cal-PAT study is an ongoing multi-centered, prospective, observational cohort study with a retrospective chart review comparison, designed to determine the effect of early administration of TXA in trauma patients with signs of hemorrhagic shock. TXA administration is currently underway in the prehospital setting (initiated March 15, 2015) and within the ED (initiated June 1, 2014). The study was started in two Southern California counties: San Bernardino and Riverside. In early 2016 Alameda County joined the study, followed by Napa County in mid-2016.

All patients ≥18 years old who have sustained blunt or penetrating trauma with signs and symptoms of hemorrhagic shock are considered for TXA treatment upon meeting the inclusion criteria (Table 1) in this ongoing study. Patients are enrolled into two prospective cohorts (known collectively as the intervention group), with a third group formed through chart-review comparison (known as the control group) (Table 2). The intervention group includes patients who received TXA and are divided into two subgroups based upon location of the administration of the first TXA dose, either prehospital intervention group or in-the-hospital intervention group. Approximately 200 patients will be enrolled in each subgroup of the intervention group for a total of 400 patients. The current study focused on comparing the prehospital intervention group with the control group. The analysis of the hospital intervention group will be discussed in future papers.

The control group consists of patients identified through chart review and has an approximate goal of 400 patients or a total that matches the combined totals of the prehospital and hospital intervention groups. Control group patients must meet the same inclusion and exclusion criteria as the intervention group and were matched based upon injury severity scores (ISS), hemodynamic profiles, and mechanism of injury. Control group patients were chosen randomly within the trauma registry of a single hospital without knowing the mortality, total blood loss, and/or side effect(s) to minimize biases and ensure data quality. Further, control group patients were transported by the same participating regional emergency medical services (EMS) agencies as intervention group patients.

TXA is administered in the prehospital setting by licensed paramedics on advanced life support (ALS) ground ambulances and registered nurses (RNs) on helicopter transport units, and in the hospital setting by licensed RNs under physician supervision. TXA is delivered in two doses following the protocol used in the CRASH-2 trial.^12^ The first dose is one gram of TXA in 100 ml of 0.9% normal saline infused over 10 minutes via intravenous or intraosseous access. It is administered as soon as possible by first responders or at participating hospitals. A green-colored wristband labeled “TXA” attached to their right wrist and/or TXA written on their chest identifies patients who receive TXA. Following arrival at a participating trauma center, patients who receive prehospital TXA are identified and re-assessed by the trauma team members for signs of hemorrhagic shock. Patients who still meet the inclusion criteria (Table 1) receive a second dose of one gram of TXA in 100 ml of 0.9% normal saline infused over eight hours via intravenous infusion.^12^ Patients who no longer meet inclusion criteria upon arrival to the hospital do not receive a second TXA dose. Patients receiving TXA in the prehospital setting make up the prehospital intervention group (Table 2).

The primary outcome of this study is mortality, measured at 24 hours, 48 hours, and 28 days. Additional outcomes include the total blood product units transfused during resuscitation efforts and during the hospital stay as well as any known adverse events associated with TXA administration such as vascular occlusive events, including deep vein thrombosis (DVT), myocardial infarction (MI) and pulmonary thromboembolism (PTE), and neurological events including stroke and seizure. Other characteristics collected include the mechanism of injury (blunt or penetrating), gender, age, and ISS.

Data were collected in San Bernardino and Riverside County. San Bernardino County consisted of 10 EMS agencies transporting to two trauma centers and Riverside County consisted of eight EMS agencies transporting to four trauma centers. The average EMS transport time by ground in San Bernardino County is approximately 22 minutes; Riverside County has similar demographic and geographic make-up.^18^ Comparable transport times may be expected in this region.

Patients in the prehospital intervention group (as opposed to the hospital intervention group) were matched with control group patients. Post-hoc analysis assessed characteristics and outcomes of patients who received one dose of TXA in comparison to two doses of TXA. A patient may have received one dose of TXA if they arrived to the trauma center and no longer satisfied inclusion criteria (Table 1). We excluded patients dead on arrival, those who received TXA for non-trauma indications, or those who were determined to be <18 years old upon arrival (Figure).

Initial patient selection (Table 1) was determined in the prehospital setting by licensed paramedics on ALS ground ambulances and RNs on helicopter transport units. Prehospital teams were educated on the inclusion and exclusion criteria of this study and had access to real-time consultation with physicians at the participating trauma centers to address any concerns in real-time regarding patient selection or TXA administration. Paramedics and RNs underwent training that included an educational film providing background on TXA, routes of administration, and known side-effect profile. This was followed by small-group educational sessions and hands-on workshops. All protocols were approved by the California Emergency Medical Services Authority (EMSA) with close supervision and oversight at both the local and state level.

### Statistical Analyses

We conducted all statistical analyses using the SAS software for Windows version 9.3 (Cary, North Carolina, USA) and R version 3.3.1. Descriptive statistics were presented as means and standard deviations for continuous variable, as well as frequencies and proportions for categorical variables. Two groups, the prehospital intervention group and the control group, were compared with regard to clinical outcomes, including 24-hour, 48-hour, and 28-day mortality, total blood product usage measured in units, and known adverse events at hospital discharge. We conducted these comparisons of clinical outcomes between the prehospital intervention and control groups using Chi-square (or Fisher’s exact test if the expected cell count <5) for categorical variables, and independent t-test for the total blood product usage. A post-hoc comparison was conducted within the prehospital intervention groups to compare the outcome between the one- and two-dose of TXA groups. We conducted a propensity score matching method using R package “MatchIt” to select patients from the control group to match the counterpart in the intervention group based on mechanism of injury, ISS and age. All statistical analyses were two-sided. We considered p-values <0.05 to be statistically significant.

## RESULTS

A total of 156 patients were identified in the original prehospital intervention group. We excluded 28 patients due to the following reasons: dead on arrival (n=4); classified as non-traumas or transferred out of the participating counties (n=19); and <18 years of age (n=5). The remaining 128 patients were included in the prehospital intervention group final analysis (see Figure for sample size flow chart). The median time for paramedics to administer TXA from the estimated time of injury was 34 minutes (interquartile range [24 min, 45 min]). More than half (59.4%, n=76) were patients who had experienced a penetrating traumatic injury, and the other 40.6% (n=52) were those who had experienced a blunt-force traumatic injury.

A total of 333 patients were identified for the original control group and included in the database. The proportion of penetrating trauma was 21.6%, which is significantly less than the proportion of penetrating in the prehospital intervention group (59.4%). To eliminate the confounding effect of mechanism of injury (blunt vs. penetrating), patients from the blunt trauma group were matched based on ISS and age with the intervention blunt group. As a result, we included a total of 53 (42.4%) blunt trauma and 72 (57.6%) penetrating trauma as the final control group (n=125). (See Figure for sample-size flow chart.)

Table 3 presents the results of comparing patients’ characteristics between the control and prehospital intervention groups. Both groups had similar percentages of penetrating trauma (control: 57.6%; intervention: 59.4%, p=0.77), similar percentages of males (control: 83.2%; intervention: 80.5%, p=0.57), and similar age (control: 39.06; intervention: 38.23, p=0.68).

Table 3 also presents the results of the comparison between clinical outcomes of the control and prehospital intervention groups. In the prehospital intervention group, use of TXA was associated with a lower 24-hour mortality rate (control: 7.2%; intervention: 3.9%, p=0.25), 48-hour mortality rate (control: 7.2%; intervention: 6.3%, p=0.76), and 28-day mortality rate (control: 10.4%; intervention: 6.3%, p=0.23), although the difference was not statistically significant. The prehospital intervention group received significantly less blood products (in units) than the control group (control: 6.95 units; intervention: 4.09 units; p=0.01), although the ISS was higher in the control group (control: 17, intervention 13; p =.0014.) Lastly, there was no significant difference in the frequency of thromboembolic events in the prehospital intervention group or control group. (Two patients with DVT prior to hospital discharge were noted in each group.) There were no PTE, MI, stroke, or seizure events recorded in either group.

We conducted a post-hoc subgroup analysis of the prehospital intervention group to identify the difference between one dose and two doses of TXA (Table 4). There was no difference with respect to the mechanism of injury, gender, age, and ISS between these two subgroups (all p-values>0.05). Regarding clinical outcomes, there was no statistically significant difference between the one-dose and two-dose prehospital subgroups regarding 24-hour mortality (one dose: 4%; two doses: 3.8%, p=0.95), 48-hour mortality (one dose: 6.7%; two doses: 5.7%, p=0.82) and 28-day mortality (one dose: 6.7%; two doses: 5.7%, p=0.82). There was no difference regarding the known adverse events at hospital discharge. The two patients with DVTs were in the two-doses subgroup. Lastly, the two-doses subgroup was administered more units of blood products (one dose: 2.45 units; two doses: 6.39 units, p=0.0079).

## DISCUSSION

The ongoing Cal-PAT study was conceived through a collaborative effort between local fire department services, first responder agencies, and multiple high-volume, university-affiliated trauma centers located throughout California. The overall goal is to assess the safety of prehospital TXA administration and impact on mortality in patients with signs of hemorrhagic shock following a traumatic injury. Initial analyses focus on the prehospital aspects of TXA administration. Hospital administration of TXA will be addressed in future analyses as the Cal-PAT study continues.

The preliminary results from the ongoing Cal-PAT study suggest that early prehospital administration of TXA may be warranted in suspected cases of trauma-related hemorrhagic shock. This study strengthens TXA literature surrounding the prehospital safety and efficacy of TXA administration through addressing short- and long-term outcomes with a larger sample size (n=128) as compared to two previous studies (n=13 and n=40).^16^,^17^ Further, initial analyses demonstrated a trend of reduced mortality with TXA administration, which was consistent with the findings of the CRASH-2 trial and MATTERs study.^12^,^15^ These results suggest that TXA may have future potential as a valuable tool for U.S. civilian EMS. To our knowledge, this is the first large-scale study to systematically examine prehospital TXA administration in the U.S.

Data trends suggest that TXA may reduce mortality at both 24 hours and 48 hours in cases of traumatic injury with signs of hemorrhagic shock. TXA is believed to exert this effect through its antifibrinolytic properties.^19^,^20^ In patients who have sustained significant blood loss, a state of fibrinolysis and hyper-fibrinolysis can be found in up to two-thirds of patients.^8^,^12^,^19^,^21^ This can threaten clot integrity and result in increased blood loss, morbidity, and mortality.^19^ TXA may act to prevent and reverse coagulopathies and reestablish hemodynamic stability. However, TXA appears to exert effect beyond 24 hours, after the risk of bleeding has decreased.^6^ The ability of TXA to decrease plasmin levels, reducing the magnitude of the pro-inflammatory effect exerted by plasmin, may be responsible for the decreased mortality observed at greater than 48 hours.^22^,^23^ Although the exact mechanism is not clear, evidence demonstrates that the therapeutic mechanism may be multifactorial in nature.

The CRASH-2 trial showed no increase in total blood products used in patients administered TXA, while the MATTERs study showed an increase in blood products used.^12^,^15^ The current study showed a statistically significant decrease in total blood product usage following TXA administration. This suggests that TXA may exert an immediate effect through its antifibrinolytic properties. Alternatively, this decreased usage of blood products observed in the current study may be attributed to a difference in injury severity between the intervention and control group, as noted by the difference in overall ISS score. It may also reflect the practice of more restrictive blood product usage observed in trauma care over the last decade.

Further, the decrease in the amount of blood products used by patients administered TXA in the Cal-PAT study could be explained by the fact that TXA was given in the prehospital setting, as opposed to the hospital upon patient arrival, as seen in the CRASH-2 trial and MATTERs study.^12^,^15^ Early administration of TXA in the prehospital setting may have allowed more time for a patient to be affected by the therapeutic effects of TXA. A post-hoc analysis of CRASH-2 data suggests that early administration of TXA to trauma patients within one hour of injury significantly reduced mortality due to hemorrhagic shock.^12^ The current study noted a <1 hour median time for paramedics to administer TXA from the estimated time of injury. Demonstrating the feasibility of rapid TXA administration by first responders is essential toward reducing the time to the first dose. Bringing TXA to the point of injury may maximize the therapeutic effect of TXA in cases of suspected trauma-induced hemorrhagic shock.^13^

Despite the proposed importance of rapid administration of TXA toward maximizing mortality benefit, administration of TXA must not delay total transport time.^13^ Emphasis in the prehospital setting should focus primarily on extrication and resuscitation. However, once peripheral intravenous access or intraosseous access is achieved, TXA can feasibly be administered. According to current U.S. EMS protocols, attempts to establish venous or intraosseous access must be made on all patients at risk for hemodynamic compromise prior to arrival at the hospital; therefore, TXA administration in the prehospital setting should not significantly increase transport time. A previous study suggested that TXA may be administered without increasing transport time.^16^ TXA is also stable at room temperature allowing for convenient storage on ambulances and helicopters. Ongoing education concerning TXA administration and indications was integrated into local and regional paramedic continuing education curriculum.

Further, TXA is an inexpensive drug that is highly cost effective. One gram of TXA, often supplied in 10ml ampules or vials, used for this study costs between $16 to $50 depending on whether TXA was purchased for prehospital or hospital use. In comparison, the raw cost for one unit of packed red blood cells is approximately $210.74, with the mean charge to the patient of $343.63.^24^ Following TXA administration, the Cal-PAT study demonstrated an approximate three-unit decrease in total blood products used; this equates to a cost reduction of approximately $500 per patient. The economic impact of TXA would be applicable across a broad spectrum of socioeconomic levels. Results from the Cal-PAT study may better elucidate EMS system characteristics within the U.S., in which TXA may confer the greatest impact.

Regarding known adverse events associated with TXA administration, we noted an equal frequency of events between the control and prehospital intervention group. This may indicate that TXA administration does not significantly increase the risk for thromboembolic events. These preliminary results are consistent with CRASH-2 trial results, but do not align with MATTERs study outcomes, which showed a slight increase in thromboembolic events in patients administered TXA.^12^,^15^ It may be noted, however, that patients included in the MATTERs study exhibited a higher injury burden, which is also associated with an increased incidence of thromboembolic events.^15^

Within the prehospital intervention group, we observed one case of a hemispheric ischemic stroke. The patient involved was a young male victim of a head-on high-energy motor vehicle collision. Upon arrival to the trauma center, physical exam showed multiple open and closed orthopedic long-bone fractures; neurological findings were unremarkable. This patient had been administered two doses of TXA per protocol. Forty hours after admission, while recovering from surgeries in the critical care unit, the patient experienced a decline in neurological status with notable fixed and dilated pupils. Repeat computed tomography (CT) of his head revealed a new large ischemic infarct with moderate mass effect, a 9mm shift, and right middle cerebral artery distribution. Suspecting traumatic vascular injury, a CT angiography (CTA) study was ordered; however, it was not completed as the family opted to instate a do-not-resuscitate (DNR) order. Without this definitive imaging study, a thromboembolic complication secondary to TXA could not be ruled out; however, it was considered remote since its relationship with respect to presentation and timing make this unlikely. The proposed cause of death in this case was vascular injury including dissection secondary to traumatic injury.

We observed a steady increase in the number of appropriate patients enrolled during the 15 months since implementation, a trend consistent with other similar studies.^8^ Correct identification of TXA candidates was an initial obstacle. Paramedics indicated that a small percentage of patients, roughly 3% (n=5) of the initial intervention group, lacked adequate identification and/or were unresponsive to questioning; as such, paramedics judged these patients’ ages based on physical appearance to be >18 years old when in fact these patients were <18 years old. We subsequently excluded these patients from analyses. Further, these events triggered immediate protocol reviews, as well as continued and repeated education for first responders arranged by EMS coordinators in each EMS agency. Additionally, real-time consultation with physicians at the participating trauma centers was available and continues to be available to paramedics in this ongoing study to aid in determining if patients meet the inclusion criteria for TXA administration. Investigators also conducted quality control within 24 hours after each case, and meetings with all hospitals and EMS agencies involved were held and continue to be held monthly to review cases and update protocols.

The literature also notes that although TXA is known to reduce blood loss in cardiopulmonary and orthopedic surgeries, the exact dosing scheme has been unclear, ranging from 2.5 to 100mg/kg and 0.25mg/kg/hr to 4mg/kg/hr for maintenance doses.^25^–^28^ Previous studies have shown no significant difference in mortality benefit between low and high doses of TXA.^29^,^30^ In emergency situations, a fixed one-gram dose followed by a one-gram maintenance dose (if a patient continued to satisfy inclusion criteria), has been deemed most practical.^12^ In the Cal-PAT study, this dosing protocol generated two prehospital subgroups (one dose vs. two doses of TXA); 58.6% of patients in the prehospital intervention group received only the first dose of TXA. This may have occurred when a patient no longer satisfied the inclusion criteria for TXA administration upon arriving at a participating trauma center, or due to lack of compliance or adherence to research protocol. Initial analyses suggested that there might be little difference in mortality between those receiving one dose versus two doses of TXA. If sufficient antifibrinolytic and anti-inflammatory effects occur with only a single dose, this challenges the apparent need for a maintenance dose. The exact half-life and duration of the maintained therapeutic level of TXA is unclear in present literature; however, reports have indicated two to three hours and approximately eight hours respectively, depending on the dosage.^31^–^33^ Further studies are warranted to clarify the optimal dosing protocol for TXA in cases of trauma-related hemorrhagic shock.

## LIMITATIONS

Initial implementation of TXA administration between the prehospital and hospital groups did not occur simultaneously (March 15, 2015, vs. June 1, 2014, respectively). The delayed onset of TXA administration in the prehospital group was due to the need for approval by local and state EMS regulatory authorities, as well as personnel training for administration in the prehospital setting. We do not believe that this difference in start date affected the quality of this study.

This study was limited by design. The prospective cohort design in comparison to a randomized control design did not allow us to administer TXA in a blinded fashion. Physicians were aware that TXA had been administered, which may have introduced a slight bias related to the level of care provided. However, we anticipate this to have minimal effect on study outcomes. The cross-matched study design and initial matching of patients by mechanism of injury further resulted in a statistically significantly greater ISS in the control group in comparison to the prehospital intervention group. We believed that mechanism of injury was most important to match; age and ISS were affected by this prioritization. As the sample size increases during this ongoing study, this discrepancy may likely be reduced. Additionally, in order to reduce biases the selection of the matched control group was random and the biostatistician did not know the outcome of interest, such as 24-hours, 48-hours, and 28-day mortality status.

Another limitation may be the difficulty associated with accurately recognizing signs of trauma-related hemorrhagic shock in the prehospital setting. High injury acuity and/or inexperience may have resulted in some EMS providers improperly including or excluding TXA candidates. As such, patients who would have qualified for this study may not have received TXA, while others who did not qualify may have received TXA. Incidences of improper exclusion were noted during the initial months after implementation and future incidences were reduced through active troubleshooting, quality control, and paramedic education. EMS teams were also backed by real-time physician consultation to provide added assistance; this teamwork approach was instituted to minimize the possibility of inappropriate TXA administration.

At the time of this report, the majority of outcomes from the Cal-PAT study do not demonstrate statistical significance. The initial conclusions presented were based upon trends; data must be interpreted with attention to this. As the sample size grows, results may have an increased likelihood of achieving statistical significance.

## CONCLUSION

Preliminary evidence from the Cal-PAT study suggests that TXA administration may be safe in the prehospital setting with no significant change in adverse events observed and an associated decreased use of blood products in cases of trauma-induced hemorrhagic shock. Given the current sample size, a statistically significant decrease in mortality was not observed. Additionally, the feasibility of prehospital identification and administration of TXA by paramedics has been demonstrated. Paramedics were able to administer TXA safely and effectively on scene and while en route to the hospital. Future continuation of data collection will enable us to explore the necessity for a second dose of TXA administered upon arrival to the hospital.

The current study indicates that TXA may be a viable option to reduce mortality in civilian prehospital trauma care within the United States. With the completion of the Cal-PAT study, we hope to further develop TXA prehospital administration protocols and support widespread implementation of TXA in the prehospital setting.

## Figures and Tables

**Figure:**
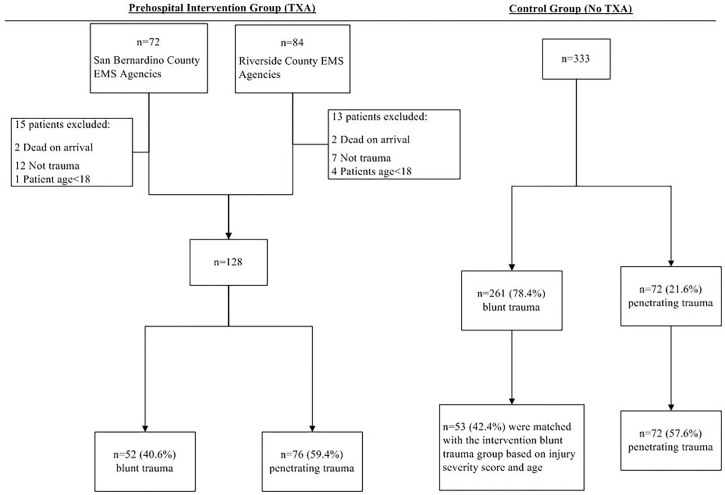
Patient exclusion flow chart that compares patient selection in the prehospital intervention group and control group. *TXA*, tranexamic acid; *EMS*, emergency medical services.

**Table 1:** Inclusion and exclusion criteria provided to first responders in the field and clinicians at receiving trauma centers, in study of efficacy of tranexamic acid (TXA) in prehospital and hospital setting. Patients receiving TXA are enrolled into the intervention group.

Inclusion criteria	Exclusion criteria
The prehospital and hospital use of TXA should be considered for all trauma patients that meet any of the following criteria: Blunt or penetrating trauma with signs and symptoms of hemorrhagic shock Systolic blood pressure of less than 90 mmHg at scene of injury, during air and/or ground medical transport, or upon arrival to designated trauma centers Any sustained blunt or penetrating injury within three hours Patients who are considered to be high risk for significant hemorrhage ○ Estimated blood loss of 500 milliliters in the field accompanied with a heart rate >120 ○ Bleeding not controlled by direct pressure or tourniquet Major amputation of any extremity above the wrists and above the ankles	Any patient <18 years of age Any patient with an active thromboembolic event (within the last 24 hours) – i.e. active stroke, myocardial infarction or pulmonary embolism Any patient with a hypersensitivity or anaphylactic reaction to TXA Any patient more than three hours post-injury Traumatic arrest with more than five minutes of cardiopulmonary resuscitation without return of vital signs Penetrating cranial injury Traumatic brain injury with brain matter exposed Isolated drowning or hanging victims Documented cervical cord injury with motor deficits

*TXA*; tranexamic acid.

**Table 2:** Classification of enrolled patients in study examining efficacy of administering TXA in prehospital vs hospital setting.

Intervention group		Control group
	
Prehospital intervention group	Hospital Intervention group	
		
Patients who received their first dose of TXA in the prehospital setting and their second dose of TXA upon arrival to the receiving trauma center (if patient continued to meet inclusion criteria).	Patients who received both doses of TXA upon arrival to the trauma center. *Data from this group was not included on the current report	Patients were chosen randomly through a chart review comparison using the trauma registry at each included hospital to identify patients with similar injury severity scores, hemodynamic profiles, and mechanism of injury to patients receiving TXA.

*TXA*; tranexamic acid.

**Table 3:** Patient outcomes comparing the control group and prehospital intervention group. The control group is matched to prehospital subgroup patients.

	Control group (n=125)	Prehospital intervention group (n=128)	p-value
Mechanism of injury			0.7745
Blunt trauma	53 (42.4%)	52 (40.6%)	
Penetrating trauma	72 (57.6%)	76 (59.4%)	
Gender			0.5733
Female	21 (16.8%)	25 (19.5%)	
Male	104 (83.2%)	103 (80.5%)	
Age, years, mean ± SD	39.06 ± 16.66	38.23 ± 15.48	0.6819
Injury severity score, mean ± SD	17 ± 10.74	12.96 ± 9.03	0.0014
Mortality at 24 hours			0.2519
Dead	9 (7.2%)	5 (3.9%)	
Mortality at 48 hours			0.7628
Dead	9 (7.2%)	8 (6.3%)	
Mortality at 28 days			0.2316
Dead	13 (10.4%)	8 (6.3%)	
Total blood products used (in units), mean ± SD	6.95 ± 9.93	4.09 ± 8.33	0.0135
Adverse events at hospital discharge**			0.6839
Deep vein thrombosis	2 (1.6%)	2 (1.6%)	
None	123 (98.4%)	126 (98.4%)	

* All percentages were column percentages. In other words, the percentages added up to 100% by column for each variable.

** The calculation of p-values for adverse event at hospital discharge was based on Fisher’s exact test.

**Table 4:** Prehospital intervention group analysis by dose(s) of TXA received. A patient may receive one dose of TXA if they no longer satisfy inclusion criteria upon arrival to a receiving trauma center.

	Prehospital 1 dose of TXA (n=75)	1 Prehospital + 1 hospital dose of TXA (n=53)	P-value
Mechanism of injury			0.8461
Blunt	31 (41.3%)	21 (39.6%)	
Penetrating	44 (58.7%)	32 (60.4%)	
Gender			0.5407
Female	16 (21.3%)	9 (17%)	
Male	59 (78.7%)	44 (83%)	
Age, years, mean ± SD	38.19 ± 16.84	38.3 ± 13.49	0.9671
ISS, mean ± SD	11.85 ± 8.43	14.53 ± 9.67	0.0989
Mortality 24 hours			0.9481
Dead	3 (4%)	2 (3.8%)	
Mortality 48 hours			0.8168
Dead	5 (6.7%)	3 (5.7%)	
Mortality 28 days			0.8168
Dead	5 (6.7%)	3 (5.7%)	
Total blood product (in units), mean ± SD	2.45 ± 6.38	6.39 ± 10.12	0.0079
Adverse event at hospital discharge**			0.1695
Deep vein thrombosis	0	2 (3.8%)	
None	75 (100%)	51 (96.2%)	

*TXA*, tranexamic acid; *EMS*, emergency medical services.

** The calculation of p-values for adverse event at hospital discharge was based on Fisher’s exact test.
